# Correction: Building customizable auto-luminescent luciferase-based reporters in plants

**DOI:** 10.7554/eLife.60938

**Published:** 2020-07-14

**Authors:** Arjun Khakhar, Colby G Starker, James C Chamness, Nayoung Lee, Sydney Stokke, Cecily Wang, Ryan Swanson, Furva Rizvi, Takato Imaizumi, Daniel F Voytas

Khakhar A, Starker CG, Chamness JC, Lee N, Stokke S, Wang C, Swanson R, Rizvi F, Imaizumi T, Voytas DF. 2020. Building customizable auto-luminescent luciferase-based reporters in plants. *eLife* **9**:e52786. doi: 10.7554/eLife.52786.

Published 25, March 2020

It has come to our attention that there are errors in Table 1, which lists the plasmids used in the study. Errors include omission of some genes in specific plasmids and the mis-identification of some promoters and terminators. In the corrected version, we also removed plasmids from Table 1 that are not used in the study. Descriptions of all plasmids in the text of the article are correct.

Additionally, we now include the number of the Department of Energy grant that helped support this work (DE-SC0018277).

The article has been corrected accordingly.

